# Exploring the associations between colorectal polyps and type 2 diabetes mellitus in a colonoscopy clinic population

**DOI:** 10.1016/j.esmogo.2024.100053

**Published:** 2024-04-15

**Authors:** J.S. Kimber, E. Symonds, W. Uylaki, M. Horsnell, P.A. Drew, E. Smith, R.R. Mikaeel, J.E. Hardingham, Y. Tomita, D. Jesudason, P.J. Hewett, W.J. Brooks, J.P. Young, T.J. Price

**Affiliations:** 1 Department of Haematology and Oncology, The Queen Elizabeth Hospital, Woodville South, Australia; 2 Faculty of Health and Medical Sciences, University of Adelaide, Adelaide, Australia; 3 Bowel Health Service, Flinders Medical Centre, Bedford Park, Australia; 4 Cancer Research, Flinders Health and Medical Research Institute, Flinders University, Bedford Park; 5 SAHMRI Colorectal Node, Basil Hetzel Institute, Woodville South, Australia; 6 Biology Department, College of Science, University of Duhok, Duhok, Kurdistan, Iraq; 7 Department of Endocrinology, The Queen Elizabeth Hospital, Woodville South, Australia; 8 Department of Surgery, The Queen Elizabeth Hospital, Woodville South, Australia; 9 Department of Adelaide Medical Solutions, Woodville South, Australia

**Keywords:** adenoma, type 2 diabetes mellitus, colorectal polyp, sessile serrated lesion

## Abstract

**Introduction:**

An association has consistently been reported between type 2 diabetes mellitus (T2DM) and colorectal cancer (CRC). CRC develops within premalignant polyps. The aim of this work was to examine the relationship between colorectal polyp subtypes and T2DM across the population, including in low- and non-screening age groups.

**Material and methods:**

A cross-sectional study was carried out using an audit of a colonoscopy database and histopathology reports at a tertiary teaching hospital during 2016. Included were consecutive patients undergoing colonoscopy for a diverse range of indications. Univariable and multivariable analyses were carried out to assess the associations between T2DM, age, sex, indications for the procedure and different types of colorectal polyps.

**Results:**

Data were extracted from colonoscopies in 1395 patients. Evidence of T2DM was observed in 257 (18%) patients. Any adenoma was present in 109 (42%) patients with T2DM compared with 256 (22%) patients with no evidence of T2DM [odds ratio (OR) 2.5, 95% confidence interval (CI) 1.9-3.4, *P* < 0.001]. In patients <50 years of age, occult bleeding was associated with any adenoma (OR 3.1, 95% CI 1.2-7.8, *P* = 0.03) and advanced adenoma (OR 21, 95% CI 1.8-240, *P* = 0.02). A multinomial logistic regression determined that male sex, T2DM, blood in the stool and older age were all independently associated with the presence of any adenoma.

**Conclusions:**

Adenomas were independently associated with T2DM. Given the consistent association between CRC and T2DM, these data suggest that a diagnosis of T2DM may warrant closer colorectal surveillance.

## Highlights

• Type 2 diabetes mellitus is associated with adenomatous colorectal polyps, the most common precursor of colorectal cancer.
• Neither hyperplastic polyps nor sessile serrated lesions were associated with type 2 diabetes mellitus.
• Occult bleeding is associated with having any adenoma, including advanced adenomas, in younger people.

## Introduction

Colorectal cancer (CRC) and type 2 diabetes mellitus (T2DM) are frequent causes of death worldwide. The American Diabetes Association has officially recognised a link between diabetes and cancer in a 2010 consensus report,^1^ and T2DM is significantly associated with risk of CRC.^2^ CRC develops from a precancerous lesion, the most common of these being the adenoma. The presence of these lesions and their relatively long dwell time renders CRC amenable to effective prevention or early diagnosis by detection and removal of these polyps, or by detection of a polyp which has newly undergone malignant transformation. A decreased incidence of CRC can be achieved through excision of adenomatous polyps^3^^,^^4^ and the process of detecting polyps and early cancers is one of the most effective methods for prevention of advanced cancer and death in any malignancy.

T2DM shows a consistent association with CRC across all age groups. A personal history of T2DM has also been linked to young-onset CRC, with the increased risk likely ranging between threefold and fourfold.^5^^,^^6^ A diagnosis of type 1 diabetes mellitus, however, appears to show no increase in risk.^6^ Of concern, a personal history of diabetes combined with a family history of CRC may raise the risk to just under seven times that of the population risk.^6^

Colorectal polyp subtypes have been associated with several lifestyle risk factors.^7^^,^^8^ Analysis of risk factors for specific types of polyps may help to identify high-risk groups in the population where specific surveillance would be of benefit.^7^ A demographic of interest in CRC, and particularly in those with T2DM, is the young-onset age group. The incidence of young-onset CRC has been increasing during the past two decades in the USA and Australia.^9^ It is important to identify risk factors for CRC and precursor lesions in the young adult population to identify potential groups for targeted screening, which could thereby achieve early detection and prevention of CRC. Young-onset CRC is associated with a personal history of T2DM,^5^ however, the relationship between T2DM and specific colorectal polyp subtypes remains unclear.

Some observational evidence suggests that most young-onset CRC are likely to arise within traditional adenomas rather than serrated lesions.^10^ Critical factors in the association between CRC and T2DM have not been accurately determined, however the high insulin environment in pre-diabetes, inflammation from the microbiota and lack of healthy lifestyle have been proposed as generating a setting of tissue repair and proliferation in which the initiation of colorectal neoplasia is more likely (reviewed in detail by Gonzalez et al.^11^). It would therefore be expected that there would also be an association between T2DM and adenomatous polyps.

The relationship between adenomas and T2DM is not clear, with some studies reporting an association with advanced lesions,^12^ but other studies reporting no association.^13^^,^^14^ This study aimed to examine the relationship between polyp subtypes and T2DM across the population. In particular the study aims to compare these associations when stratified into both low- and non-screening (age <55 years) and older (age ≥55 years) age groups, as T2DM may have the potential to serve as a neoplasia associated risk marker in younger adults. We also aimed to discover if specific indications for colonoscopy predicted these outcomes to detect practical implications of potential associations.

## Material and methods

This was a cross-sectional study using an audit of procedures recorded within the colonoscopy database at a tertiary teaching hospital^15^ over a 6-month period from January 2016. Data from each procedure were recorded in sequence in a purpose-dedicated database. Findings were obtained from both the colonoscopy report within a ProVation endoscopy procedure documentation system (Wolters Klewer, USA)^16^ and the corresponding pathology report concerning histological examination of excised lesions. T2DM status was determined from medical records. Patients in this study were partitioned into low- and non-screening individuals (aged <55 years), and those ≥55 years. Though population screening for CRC in Australia begins at age 50 years, the rationale for this study’s partition of the groups is as follows: low rates of population screening uptake in the overlapping age group (50-54 years of age),^17^ the more pronounced risk of CRC in patients aged <55 years who develop T2DM and reported increasing mortality in patients aged <55 years in the USA.^18^, ^19^, ^20^ Included were data from each consecutive colonoscopy carried out within this period at this hospital that did not meet exclusion criteria. Excluded were the patients and procedures with incomplete data, abandonment of procedure, poor preparation necessitating a scheduled repeat procedure, or repeat procedures within the study period. The colonoscopies were carried out by trained physicians and surgeons. The study was approved by the Human Research Ethics Committee of the Central Adelaide Local Health Network under protocol number HREC/14/TQEHLMH/194.

### Definitions

Lesion subtypes were reported on a ‘per colonoscopy’ basis. Serrated lesions included hyperplastic polyps and sessile serrated lesions (formerly known as sessile serrated adenoma/polyps).^21^ ‘Any adenoma’ comprised conventional adenomas with tubular and/or villous histology and low- or high-grade dysplasia. Villous adenomas and traditional serrated adenomas were rare, therefore described in narrative form however not included in the statistical analyses individually.^22^ ‘Advanced adenomas’ were defined as those with at least one of: high-grade dysplasia, diameter ≥10 mm, or villous histology.^23^^,^^24^ T2DM status and indication for colonoscopy were discerned from medical records documentation. ‘Overt’ bleeding was that presenting as a symptom. ‘Occult bleeding’ was that diagnosed from a faecal blood test rather than as a symptom. The study size was determined by the number of cases that were carried out during the time of the audit.

### Statistical analysis

Personal characteristics, indications for colonoscopy and findings were compared between two groups (those with and without evidence of T2DM). In addition, associations were explored between potential confounders such as age, sex, indications for the procedure and findings at colonoscopy. Associations were analysed using Pearson’s chi-square test. Multinomial logistic regression for multivariable analysis included all associations with *P* values <0.20. All statistical association tests were carried out using SPSS version 26 for Mac (IBM, USA). Two-tailed statistics were used throughout with a significance level of *P* value <0.05.

## Results

Of the total 1657 available procedures, 262 (16%) met the exclusion criteria. The analysis was carried out on the remaining 1395. Males comprised 50% (691) of this total, those aged <50 years encompassed 24% (340) and 18% (257) of all patients had T2DM. Of patients with T2DM, 54% (140) were male and 6% (15) were under the age of 55 years. Any polyp was present in 487 cases (35%), with adenoma in 365 (26%), sessile serrated lesion in 72 (5%) and hyperplastic polyp in 140 (10%). Two villous adenomas and no traditional serrated adenomas were found. Indications for colonoscopy were available in 1278 patients (92%) and were considered as dichotomous variables as follows: surveillance for higher risk conditions (e.g. previous neoplasia, inflammatory bowel disease), family history of CRC and any bleeding (which was partitioned into occult and overt bleeding). The adenoma detection rate in eligible patients (those aged ≥50 years without inflammatory bowel disease) in this cohort was 32%, which is above the rate for standard of practice in Australia.^25^ Characteristics of patients are shown in Table 1.

**Table 1:** Patient characteristics

Total number of patients		1395
Age in years (median, mean)		60, 58.9
Age groups, *n* (%)	<50	340 (24)
	≥50	1055 (76)
Male sex, *n* (%)		691 (50)
Type 2 diabetes, *n* (%)	Total	257 (18)
	Male sex	140 (54)
	Aged <50	15 (6)
Polyp subtypes, *n* (%)	Any adenoma	365 (35)
	Tubular adenoma	337 (24)
	Tubular adenoma with high grade dysplasia	11 (3)
	Tubulovillous adenoma	54 (4)
	Villous adenoma	2 (0.1)
	Advanced adenoma	86 (6)
	Hyperplastic polyp	140 (10)
	Sessile serrated lesion	72 (6)
Indications for colonoscopy^a^, *n* (%)	Surveillance	353 (28)
	Family history reported	97 (8)
	Any bleeding	501 (39)
	Overt bleeding	206 (16)
	Occult bleeding	295 (23)

a Indications available for 1278 cases, with some categories overlapping.

Univariable analyses are demonstrated in Table 2. Being aged ≥50 years was significantly associated with any conventional adenoma (odds ratio (OR) 0.3, 95% confidence interval (CI) 0.2-0.4, *P* < 0.001), tubular adenoma (OR 0.3, 95% CI 0.2-0.4, *P* < 0.001), tubulovillous adenoma (OR 0.2, 95% CI 0.1-0.6, *P* = 0.004), any advanced adenoma (OR 0.1, 95% CI 0.1-0.4, *P* < 0.001) compared with those aged <50 years. Hyperplastic polyps and sessile serrated lesions showed no relationship with age group. Male sex was significantly associated with any conventional adenoma (OR 1.6, 95% CI 1.2-2.0, *P* < 0.001), tubular adenoma (OR 1.6, 95% CI 1.3-2.1, *P* < 0.001), hyperplastic polyps (OR 1.7, 95% CI 1.2-2.4, *P* = 0.003) and any advanced adenoma (OR 1.6, 95% CI 1.1-2.3, *P* = 0.03). Sessile serrated lesions and tubulovillous adenomas were not associated with sex. Family history of CRC and surveillance as indications for colonoscopy were not significantly associated with any polyp subtype.

**Table 2:** Patient characteristics and polyp subtype

Variable		Any adenoma, *n* (%)	Tubular adenoma, *n* (%)	Tubulovillous adenoma, *n* (%)	Advanced adenoma, *n* (%)	Hyperplastic polyp, *n* (%)	Sessile serrated lesion, *n* (%)
**Type 2 diabetes**	Diabetics (*n* = 257)	109 (42)	100 (39)	16 (6)	28 (11)	34 (13)	15 (6)
	Non-diabetics (*n* = 1138)	256 (22)	237 (21)	38 (3)	58 (5)	106 (9)	57 (5)
*P* value		**<0.001**	**<0.001**	**0.003**	**<0.001**	0.06	0.59
OR (95% CI)		2.5 (1.9-3.4)	2.4 (1.8-3.2)	1.9 (1.1-3.5)	2.2 (1.4-3.7)	1.4 (1.0-2.2)	1.2 (0.7-2.1)
**Age group**	<50 years (*n* = 340)	38 (11)	36 (11)	3 (1)	4 (1)	26 (8)	17 (5)
	≥50 years (*n* = 1055)	327 (31)	301 (29)	51 (5)	82 (8)	114 (11)	55 (5.2)
*P* value		**<0.001**	**<0.001**	**0.004**	**<0.001**	0.09	0.88
OR (95% CI)		0.3 (0.2-0.4)	0.3 (0.2-0.4)	0.2 (0.1-0.6)	0.1 (0.1-0.4)	0.7 (0.4-1.1)	0.95 (0.5-1.7)
**Sex**	Males (*n* = 691)	211 (31)	197 (29)	30 (4)	53 (8)	86 (12)	37 (5)
	Females (*n* = 704)	153 (22)	139 (20)	24 (3)	33 (5)	54 (8)	35 (5)
*P* value		**<0.001**	**<0.001**	0.37	**0.02**	**0.003**	0.67
OR (95% CI)		1.6 (1.2-2.0)	1.6 (1.3-2.1)	1.3 (0.7-2.2)	1.6 (1.1-2.3)	1.7 (1.2-2.4)	1.1 (0.7-1.7)
**Family history CRC**	Family History (*n* = 97)	22 (23)	20 (21)	3 (3)	7 (7)	12 (12)	5 (5)
	No family history (*n* = 1181)	308 (26)	286 (24)	48 (4)	96 (8)	116 (10)	58 (5)
*P* value		0.46	0.43	0.64	0.75	0.42	0.9
OR (95% CI)		0.8 (0.5-1.4)	0.8 (0.5-1.3)	0.8 (0.2-2.4)	0.9 (0.4-2.0)	1.3 (0.7-2.4)	1.0 (0.4-2.6)
**Surveillance**	Surveillance (*n* = 353)	94 (27)	87 (25)	14 (4)	28 (8)	36 (10)	19 (5)
	Not under surveillance (*n* = 925)	236 (26)	219 (24)	37 (4)	75 (8)	92 (10)	44 (5)
*P* value		0.68	0.72	0.98	0.92	0.89	0.64
OR (95% CI)		1.0 (0.8-1.4)	1.1 (0.8-1.4)	1.0 (0.5-1.9)	0.9 (0.6-1.5)	1.0 (0.7-1.5)	1.1 (0.7-2.0)
**Any bleeding**	Bleeding (*n* = 501)	151 (30)	142 (28)	24 (5)	38 (9)	56 (11)	25 (5)
	No bleeding (*n* = 777)	179 (23)	164 (21)	27 (4)	42 (7)	72 (9)	38 (5)
*P* value		**0.005**	**0.003**	0.24	0.12	0.27	0.94
OR (95% CI)		1.4 (1.1-1.8)	1.5 (1.1-1.9)	1.4 (0.8-2.4)	1.4 (0.9-2.3)	1.2 (0.9-1.8)	1.0 (0.6-1.7)
**Overt bleeding**	Overt bleeding (*n* = 206)	43 (20)	40 (19)	5 (3)	10 (5)	22 (11)	9 (4)
	No overt bleeding (*n* = 1069)	287 (27)	266 (25)	46 (42)	70 (7)	106 (10)	54 (5)
*P* value		**0.039**	**0.05**	0.44	0.35	0.8	0.73
OR (95% CI)		0.7 (0.5-0.9)	0.7 (0.5-1.1)	0.7 (0.3-1.6)	0.7 (0.4-1.4)	1.1 (0.7-1.7)	0.8 (0.4-1.7)
**Occult bleeding**	Occult bleeding (*n* = 295)	108 (37)	102 (35)	19 (6)	28 (10)	34 (12)	18 (5)
	No occult bleeding (*n* = 935)	222 (24)	204 (21)	32 (3)	52 (5)	94 (10)	45 (5)
*P* value		**<0.001**	**<0.001**	**0.01**	**0.009**	0.46	0.77
OR (95% CI)		1.9 (1.4-2.5)	1.9 (1.5-2.5)	2.0 (1.1-3.5)	1.8 (1.2-3.0)	1.2 (0.8-1.7)	1.1 (0.6-1.9)

CRC, colorectal cancer; CI, confidence interval; OR, odds ratio.

Results highlighted in bold if *P* is equal to or less than 0.05.

Any adenomatous polyp was present in 109 (42%) patients with T2DM compared with 148 (13%) patients with no evidence of T2DM (OR 2.5, 95% CI 1.9-3.4, *P* < 0.001). T2DM was specifically associated with tubular adenoma (OR 2.4, 95% CI 1.8-3.2, *P* < 0.001), tubulovillous adenoma (OR 1.9, 95% CI 1.1-3.5, *P* = 0.003) and advanced adenoma (OR 2.2, 95% CI 1.4-3.7, *P* < 0.001). Neither hyperplastic polyps nor sessile serrated lesions showed a significant association with T2DM.

Patients with any bleeding were more likely to have adenomas (OR 1.4, 95% CI 1.1-1.8, *P* = 0.005) and tubular adenomas (OR 1.5, 95% CI 1.1-1.9, *P* = 0.003), but not tubulovillous adenomas, hyperplastic polyps or sessile serrated lesions. Occult bleeding was associated with adenomas (OR 1.9, 95% CI 1.4-2.5, *P* < 0.001), tubular adenomas (OR 1.9, 95% CI 1.5-2.5, *P* < 0.001), tubulovillous adenomas (OR 2.0, 95% CI 1.1-3.5, *P* = 0.02) and advanced adenomas (OR 1.8, 95% CI 1.2-3.0, *P* = 0.009). Overt bleeding was negatively associated with any adenoma (OR 0.7, 95% CI 0.5-0.9, *P* = 0.039) and tubular adenoma (OR 0.7, 95% CI 0.5-1.1, *P* = 0.05).

When partitioned for age group, association with T2DM did not persist for any adenoma and tubular adenoma in those aged <50 years. Advanced adenomas were associated with T2DM in those aged ≥50 years (OR 1.7, 95% CI 1.1-2.8, *P* = 0.03), although this association did not persist in patients aged <50 years. T2DM was also associated with any adenoma (OR 2.1, 95% CI 1.5-2.8, *P* < 0.001) and tubular adenoma (OR 2.0, 95% CI 1.5-2.8, *P* < 0.001) in those with age ≥50 years (Table 3).

**Table 3:** Type 2 diabetes and polyp type by age

Age <50 years	Type 2 diabetes	No evidence of Type 2 diabetes	*P* value	OR	95% CI
	(*n* = 15)	(*n* = 325)			
Any adenoma, *n* (%)	3 (20)	35 (11)	0.23	2.1	0.6-7.7
Tubular adenoma, *n* (%)	2 (13)	34 (10)	0.67	1.3	0.3-6.0
Tubulovillous adenoma, *n* (%)	1 (6)	2 (0.09)	0.14	11.5	0.98-134
Any advanced adenoma, *n* (%)	1(6)	3 (0.9)	0.17	7.6	0.7-78

Age ≥50 years	Type 2 diabetes	No evidence of Type 2 diabetes	*P* value	OR	95% CI
	(*n* = 242)	(*n* = 658)			
Any adenoma, *n* (%)	106 (43)	221 (34)	<0.001	2.1	1.5-2.8
Tubular adenoma, *n* (%)	98(40)	203 (31)	<0.001	2	1.5-2.8
Tubulovillous adenoma, *n* (%)	15 (6)	36 (5)	0.30	1.4	0.8-2.6
Any advanced adenoma, *n* (%)	27 (11)	55 (8)	0.03	1.7	1.1-2.8

CI, confidence interval; OR, odds ratio.

The association between any adenoma and occult bleeding persisted in patients <50 years of age (OR 3.1, 95% CI 1.2-7.8, *P* = 0.03) and in those ≥50 years (OR 1.6, 95% CI 1.1-2.1, *P* = 0.003). The association between advanced adenomas and occult bleeding remained for those aged <50 years (OR 21, 95% CI 1.8-240, *P* = 0.02) but not for those ≥50 years of age (Table 4).

**Table 4:** Bleeding and any adenoma by age

Age <50 years	Occult bleeding	No occult bleeding	*P* value	OR	95% CI
	(*n* = 29)	(*n* = 286)			
Any adenoma, *n* (%)	7 (24)	27 (9)	0.025	3.1	1.2-7.8
Any advanced adenoma, *n* (%)	2 (7)	1 (0.3)	0.023	21	1.8-240

Age ≥50 years	Occult bleeding	No occult bleeding	*P* value	OR	95% CI
	(*n* = 266)	(*n* = 698)			
Any adenoma, *n* (%)	101 (37)	195 (28)	0.003	1.6	1.1-2.1
Any advanced adenoma, *n* (%)	26(10)	51 (7.3)	0.23	1.3	0.8-2.3

CI, confidence interval; OR, odds ratio.

A multivariable logistic regression was carried out which showed that female sex (OR 0.6, 95% CI 0.46-0.79, *P* = 0.001), age ≥50 years (OR 2.1, 95% CI 1.6-2.8, *P* < 0.001), occult bleeding (OR 1.9, 95% CI 1.2-2.5, *P* = 0.003) and T2DM (OR 2.2, 95% CI 1.5-2.8, *P* < 0.001) were each independently associated with the presence of any adenomatous polyp. Advanced adenoma was also independently associated with female sex (OR 0.6, 95% CI 0.37-0.95, *P* = 0.03), age ≥50 years (OR 7.3, 95% CI 2.2-23.6, *P ≤* 0.001) and T2DM (OR 1.6, 95% CI 1.0-2.6, *P* = 0.04), but not occult bleeding or any bleeding (Table 5).

**Table 5:** Adenoma and advanced adenoma by demographics

Any adenoma	*P* value	OR	95% CI
Type 2 diabetes	<0.001	2.2	1.5-2.8
Male sex	0.001	0.6	0.46-0.79
Age ≥50 years	<0.001	2.1	1.6-2.8
Occult bleeding	0.003	1.9	1.2-2.5
Any bleeding	0.85	1.1	0.7-1.4

Advanced adenoma	*P* value	OR	95% CI
Type 2 diabetes	0.03	1.8	1.1-2.6
Male sex	0.03	0.6	0.37-0.95
Age ≥50 years	<0.001	7.3	2.2-23.6
Occult bleeding	0.18	1.7	0.8-2.6
Any bleeding	0.98	1.0	0.5-2.0

CI, confidence interval; OR, odds ratio.

The trend seen in univariable analysis for hyperplastic polyps to be associated with T2DM was not present (OR 1.3, 95% CI 0.9-2.0, *P* = 0.22) when adjusted for confounding by age (OR 1.4, 95% CI 0.9-2.1, *P* = 0.09). Male sex remained an independent predictor of hyperplastic polyps (OR 1.6, 95% CI 1.1-2.5, *P* = 0.02) when controlled for the other demographics measured.

## Discussion

In this study, adenomatous polyps were shown to be associated with T2DM, with twofold the number of patients with T2DM having adenomas found compared with the incidence in those without T2DM. This finding suggests that a personal history of T2DM increases the risk of developing adenomatous polyps, the most common precursor of CRC. Of note, advanced adenoma subtypes, as well as tubulovillous adenomas, a subset with higher risk for malignant transformation, also showed an association with T2DM. The association between T2DM and tubular adenoma did not persist in the younger age group, however the statistical power was low. This association did persist in the older age group, as well as the association of T2DM with any adenoma and advanced adenoma. Of note, our data found that neither hyperplastic polyps nor sessile serrated lesions were associated with T2DM. A colonoscopy study of patients aged 40-49 years with and without diabetes in the USA during 2014 found a threefold increase in risk for adenomatous polyps in those with diabetes.^26^ Two studies, using molecular^27^ and histopathology^10^ features have suggested that adenomas are the most likely precursor lesions for young-onset CRC. Our findings support these data.

This study also highlighted features of sessile serrated lesions. These polyps showed no relationship with age group, sex or T2DM. The independence of age group for these lesions adds evidence for the theorised prolonged dwell time of these lesions.^28^ In addition, hyperplastic polyps and sessile serrated lesions were not associated with any bleeding, occult or overt, as an indication for colonoscopy. This appears to be consistent with findings of previous studies.^10^^,^^29^

Occult bleeding was associated with having both any adenoma and advanced adenoma in the young age group. This would suggest that the use of occult bleeding screening tests (e.g. faecal immunochemical tests) may be useful in this group to identify those at higher risk, as young adults are not eligible for routine population screening.

The association between T2DM and CRC, independent of their shared risk factors, may be contributed to by hyperinsulinaemia, hyperglycaemia and antidiabetic medications, as suggested by observational evidence.^1^^,^^30^ Growth factor and antiapoptotic properties are exhibited by insulin and insulin-like growth factor-1. High glucose levels and advanced glycation end products induce oxidative stress and inflammation and increase proliferation of cultured colon cancer cells.^31^^,^^32^ These among other mechanisms appear likely to promote conditions for cancer growth.^11^

The study has several limitations, some of which reflect real-world practice. Patients included underwent procedures for a diverse range of indications. In addition, the population did not include cases of colorectal polyps diagnosed by sigmoidoscopy, and it is difficult to know whether this has influenced the ability to generalise to all patients undergoing investigations. Procedures were carried out in a tertiary teaching hospital by multiple colonoscopists and lesions excised underwent a single pathology review. The adenoma detection rate for the colonoscopy unit was 32% which is within acceptable limits required for individual accreditation in Australia and is consistent with published levels from international societies.^33^

A further limitation of our study is that T2DM status was extracted from patient records, rather than systematically assessed. The prevalence of T2DM in our colonoscopy population audit is considerably higher at 18.4% than that estimated for Australian adults of 6%.^34^ It appears likely, however, that T2DM prevalence is greater in the colonoscopy clinic demographic. A similar prevalence of T2DM was seen in the colonoscopy population of a public hospital in the northern suburbs of Adelaide, where 291 patients underwent a purpose-built questionnaire before their procedure, with 18.8% of male and 21.4% of female adults reporting having diabetes.^14^ As T2DM is often asymptomatic, our figures may have underestimated the prevalence due to sample migration. Our study also did not have information on T2DM control in the affected patients. A recent report from the USA has suggested, however, that neither duration nor type of treatment of T2DM showed a significant association with adenomatous polyps.^13^ Whether the colonoscopy included was the first or subsequent procedure for a given patient was not included in the data, a further limitation. In addition, some stratified analyses in our study may have lacked adequate power to reveal true associations due to low sample sizes.

Several previously associated risk factors for adenomas, specifically body mass index and smoking, were not able to be examined in this audit due to incompleteness of available data. Hu et al.^35^ demonstrated an independent association between T2DM and CRC in a large study of 201 061 person-years of follow-up, after adjustment for age, BMI and activity level suggesting that T2DM is the likely driver for the association. The association between smoking and colorectal adenomas has been shown to be significantly affected by the duration of the exposure.^36^^,^^37^ Its effects on the adenoma detection rate, however, as well as that of BMI, have recently been shown to be independent of that associated with T2DM.^38^ The incomplete data available for family history of CRC in our study may have contributed to limited ability of this variable to predict polyp subtypes. This parameter was suggested to be underreported in a recent study, which identified family history as a forgotten question in routine clinical practice.^39^ As this was carried out at a single tertiary centre with an ethnically diverse population and on a reasonable sized sample group, our findings likely have fair generalisability. Given the time frame of the data collection in 2016, at time of publication the generalisability of the results to modern endoscopy and polyp detection techniques may also be limited.

The prevalence of T2DM increases with age but there are worrying trends for an increasingly young age at diagnosis.^40^ It has been observed recently that if T2DM is diagnosed before age 50 years, CRC is diagnosed at a median age of 59 years compared with the population median of 71 years.^6^ The time between development of an adenoma and malignant transformation is likely >10 years,^41^, ^42^, ^43^ hence early screening for CRC in patients who develop T2DM before the age of 50 years would then appear to be justified. Our data support the building evidence that early-onset T2DM may be another factor which could assist to identify younger adults who could benefit from earlier CRC screening. In addition, our data further suggest that using faecal immunochemistry testing as a screening method may be effective in identifying those young adults with T2DM at increased risk of CRC.
